# Research collaboration initiative

**DOI:** 10.1016/j.archoralbio.2009.07.001

**Published:** 2009-12

**Authors:** Alan Brook

**Affiliations:** University of Liverpool, School of Dental Sciences, Edwards Building, Daulby Street, Liverpool L69 3GN, United Kingdom

This special supplement to the Archives of Oral Biology reflects a collaborative initiative within the area of oro-facial growth and development. A Workshop was held in Liverpool in November 2007 to establish an International Collaborating Centre in Oro-facial Genetics and Development. This issue contains papers from that Workshop refereed by colleagues from the wider dental community.

In bringing this group of colleagues together we have been inspired by the statue in Lisbon (Fig. 1) which celebrates the discoveries of the Portuguese navigators of the fifteenth century. As this memorial celebrates those advances were only possible because of the collaboration and teamwork of numerous skilled individuals from many backgrounds. The International Collaborating Centre seeks to bring together colleagues from different centres around the world who are using different approaches. We are seeking to build on existing collaborations, such as the Wellcome Programme led by Jennifer Kirkham and the Royal Society of Medicine Visiting Professorships for Alan Brook and Tim Wright. The Workshop was an initial step towards this goal, and the members of the Collaborative Centre are eager for other colleagues to join who feel their research may benefit from the joint approaches available.

As a basis for that future development this issue contains a series of papers which are either critical perspectives on specific topics or which report new studies in the area.

The initial group of papers sets the scene. The first paper considers the multifactorial, multilevel, multidimensional and time related interactions which occur during normal and abnormal dental development. In the second paper a particular aspect of the multifactorial effects, the role of human sex chromosomes in dental development is reviewed while from the multilevel effects the third paper focuses on epithelial histogenesis during tooth development. This initial overview section closes with a consideration of multidimensional and time dependant effects by developing a current, clinically relevant update of the morphogenetic fields concept, incorporating a synthesis of the clone theory and the odontogenic homeobox code.

The next section of papers considers the multifactorial effects on the initiation and morphogenesis phases of dental development. It begins with a critical evaluation of studies involving twins to determine genetic and environmental influences on human dental development. There follows a series of new studies on variation and anomalies of human tooth number, size and shape. These begin with family studies, first on the aetiology of hypodontia and then on the tooth dimensions in a family with hypodontia associated with an identified PAX 9 mutation. The contrasting tooth dimensions in patients with hypodontia and supernumerary teeth compared with controls are examined in the next study. There follows a paper reporting the detailed investigation of multiple crown size variables of upper incisor teeth in patients with supernumerary teeth compared with controls. This group of new studies concludes with the examination of variability and patterning of permanent tooth size in four human ethnic groups.

The third group of papers consists of three new studies on the differentiation and biomineralisation phases of human dental development. The first of these examines enamel defects in extracted and exfoliated teeth from patients with Amelogenisis Imperfecta using the extended Enamel Defect Index and image analysis. This is followed by a study of the patterns of enamel hypoplastic defects in two archaeological populations and the section concludes with an investigation of demineralisation rates in pre-natal, neo-natal and post-natal enamel.

The fourth section considers first the crucial role of critical evaluation of measurement studies of the phenotype resulting from the dental developmental process. It concludes with a consideration of innovative 3D methodologies to allow more detailed definition of dental phenotypes in order to enhance discrimination between individuals and to provide further insight into the aetiology of dental anomalies in the future.

I am grateful to several people for their input to the preparation of this issue. Rebecca Griffin has been highly efficient and supportive as Assistant Guest Editor. Karen Barnes provided substantial and valuable administrative support in the organisation of the International Workshop. The Editor-in-Chief of the Archives of Oral Biology, Rex Holland, has given firm and clear guidance on the editorial process and has carefully and appropriately overseen the refereeing process. On the production side Nick Dunwell, Account Executive for Elsevier Pharma Solutions has been helpful and encouraging.

This issue both makes available papers from the International Workshop to the wider research community and acts as a basis for future collaborative work. It is made possible by generous financial support from the Wellcome Trust, for those papers arising from the Wellcome Programme on Biomineralisation, from Boots plc and from the University of Liverpool. The Workshop was funded by donations from Boots plc, the Universities of Adelaide and Liverpool and Scantron Ltd. We are most grateful for this generous support.

## Figures and Tables

**Fig. 1:**
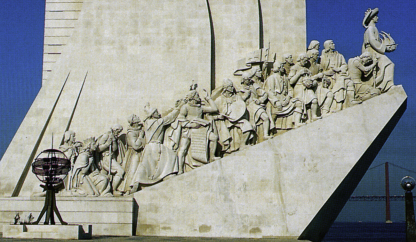
Prince Henry the Navigator holding a caravel. Behind him are cartographers, mathematicians, astronomers, carpenters, cordwainers, shield bearers, pilots, captains, doctors, zoologists, missionaries, and chroniclers – all those who took part in the epic of the Discoveries.

